# Identification of gene co-regulatory modules and associated *cis-*elements involved in degenerative heart disease

**DOI:** 10.1186/1755-8794-2-31

**Published:** 2009-05-28

**Authors:** Charles G Danko, Arkady M Pertsov

**Affiliations:** 1 Department of Pharmacology, SUNY Upstate Medical University, Syracuse, NY, USA

## Abstract

**Background:**

Cardiomyopathies, degenerative diseases of cardiac muscle, are among the leading causes of death in the developed world. Microarray studies of cardiomyopathies have identified up to several hundred genes that significantly alter their expression patterns as the disease progresses. However, the regulatory mechanisms driving these changes, in particular the networks of transcription factors involved, remain poorly understood. Our goals are (A) to identify modules of co-regulated genes that undergo similar changes in expression in various types of cardiomyopathies, and (B) to reveal the specific pattern of transcription factor binding sites, *cis*-elements, in the proximal promoter region of genes comprising such modules.

**Methods:**

We analyzed 149 microarray samples from human hypertrophic and dilated cardiomyopathies of various etiologies. Hierarchical clustering and Gene Ontology annotations were applied to identify modules enriched in genes with highly correlated expression and a similar physiological function. To discover motifs that may underly changes in expression, we used the promoter regions for genes in three of the most interesting modules as input to motif discovery algorithms. The resulting motifs were used to construct a probabilistic model predictive of changes in expression across different cardiomyopathies.

**Results:**

We found that three modules with the highest degree of functional enrichment contain genes involved in myocardial contraction (n = 9), energy generation (n = 20), or protein translation (n = 20). Using motif discovery tools revealed that genes in the contractile module were found to contain a TATA-box followed by a CACC-box, and are depleted in other GC-rich motifs; whereas genes in the translation module contain a pyrimidine-rich initiator, Elk-1, SP-1, and a novel motif with a GCGC core. Using a naïve Bayes classifier revealed that patterns of motifs are statistically predictive of expression patterns, with odds ratios of 2.7 (contractile), 1.9 (energy generation), and 5.5 (protein translation).

**Conclusion:**

We identified patterns comprised of putative *cis*-regulatory motifs enriched in the upstream promoter sequence of genes that undergo similar changes in expression secondary to cardiomyopathies of various etiologies. Our analysis is a first step towards understanding transcription factor networks that are active in regulating gene expression during degenerative heart disease.

## Background

Heart disease is the leading cause of death in the developed world. Chronic heart disease is usually associated with tissue remodeling that induces maladaptive changes in gene expression and the cellular composition of cardiac tissue. Different forms of the disease are widely believed to progress according to distinct programs of gene expression that converge in end stage heart failure to similar phenotypes [1]. Microarrays have been used to characterize these differences, typically by focusing on changes in gene expression that exceed a statistical threshold [2,3]. Such methods of gene selection have proven useful for classifying different etiologies [4,5] and explaining certain aspects of the pathophysiology [6-9]. However, such a strategy is not able to identify the network of regulatory factors that facilitate gene expression in healthy tissue and during cardiac disease. In the present study, we apply a set of basic analytical tools to identify regulatory factors using microarray data and the upstream promoter sequence of each gene. We apply these tools to predict *cis*-regulatory motifs involved in remodeling cardiac tissue in different types of human cardiomyopathy.

It is well established in yeast [10] and cultured human cells [11] that genes involved in a common physiological function tend to be regulated as groups. In such a group, often called a co-regulatory module [12], genes undergo similar changes in expression that act to roughly preserve their expression ratio over different physiological conditions and intrinsic genetic cues. Our goal is to identify such modules in human cardiomyopathies, under the assumption that these modules can provide information about the regulatory factors that control expression. Our analysis uses publicly available microarray data for human ventricular tissue remodeling due to a variety of cardiac disease states. To identify likely regulatory modules in this data, we applied a hierarchical clustering algorithm to the Pearson correlation between gene expression levels across the different cardiomyopathies. Resulting clusters were visualized and characterized based on Gene Ontology annotations for function. With this analysis, we identified 35 modules, the largest of which are enriched in genes whose primary function is related to energy generation or protein translation.

Next, we addressed the question of what controls the coordinated changes in gene expression that are observed during heart disease. It is well accepted that changes in gene expression are encoded by the combinatorial activity of several different transcription factor proteins working in concert [13-15]. Changes over different physiological conditions presumably involve the activity of different combinations of transcription factors; genes whose expression is controlled by the same set of transcription factors may be expected to undergo similar changes in expression [15]. Transcription factors associated with the regulation of a gene can be identified by the presence of characteristic *cis*-regulatory motifs in the upstream promoter sequence to which they bind. Therefore, we sought to identify putative regulatory motifs involved in transcriptional regulation of genes composing the different co-regulatory modules. Our motif discovery strategy identified 17 motifs, and we validated their function with additional bioinformatic analysis using other genes.

## Methods

### Microarray Data Normalization and Batch Effect Correction

**Figure 1:**
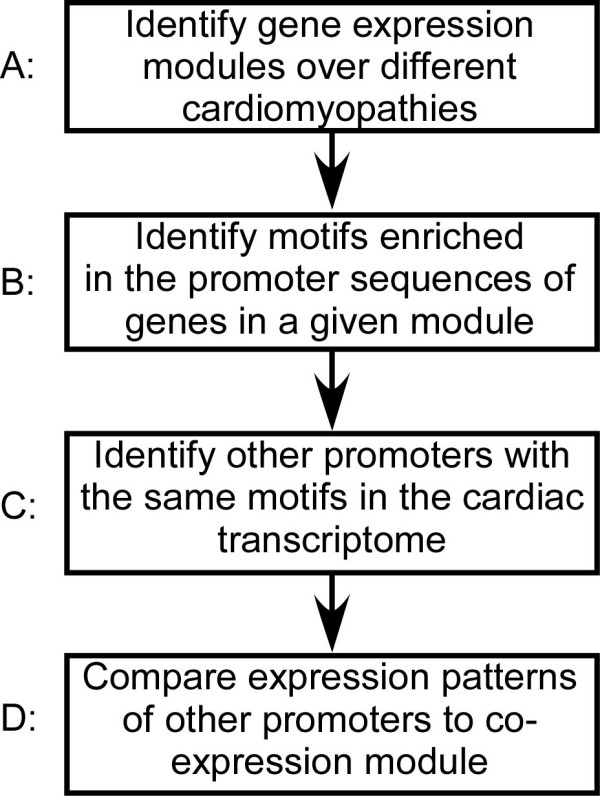
**Flowchart for identifying and testing putative motifs that affect gene transcription in cardiac disease**.

The first step in our basic experimental plan (outlined in Figure 1) was to identify genes that are co-expressed across the spectrum of different heart diseases. All ventricular microarray experiments based on the Affymetrix U133A or U133 2.0 platform for which raw CEL files were available, were collected from Gene Expression Omnibus [16] and Harvard's Cardiogenomics website (http://www.cardiogenomics.org, Jan 2007). CEL files for the U133A and U133 2.0 platform were RMA normalized separately using Bioconductor's Affy package [17]. Data sets were combined by dropping probesets not present in the U133A array from the U133 2.0 data. To correct for batch effects we used a recently described method [18]. First, samples were grouped categorically by biological condition and lab of origin, as in Figure 1. The same category of "normal ventricle" was assigned to control samples from the GSE1145, GSE2240, and GSE3585 datasets, and the category "ischemic cardiomyopathy" was assigned to the corresponding samples from GSE1145 and GSE974 datasets. All other samples were assigned a separate category. Next, the Empirical Bayes batch effect correction method [18] was applied using an R script kindly provided on the authors' website. We dropped one outlier from the final table (GSM14948) because we noted abnormally low expression of many cardiac genes, potentially indicating changes in the cellular composition of the tissue.

We verified the effectiveness of batch-effect correction using hierarchical clustering of the 149 different conditions. Whereas prior to the correction of batch effects, hierarchical clustering grouped samples by different labs, after correction samples were not grouped together (not shown). We also ensured that our correction preserved legitimate biological variation by training a naïve Bayes which classifier correctly classified the disease type with 75% accuracy (not shown). The resulting data table was a matrix with rows representing each probe in the Affymetrix U133A platform, and columns representing each of the 149 individual microarray samples.

The mean and variance of each probe was calculated for each of the 16 biological conditions described in Additional file 1, leaving a data table with rows representing each of the Affymetrix U133A probesets and 16 columns corresponding to each biological condition. This data table was used for subsequent analysis, and is referred to below as the "cardiomyopathy data".

### Creating Pairwise Correlations and Assembling the Correlation Matrix

**Figure 2:**
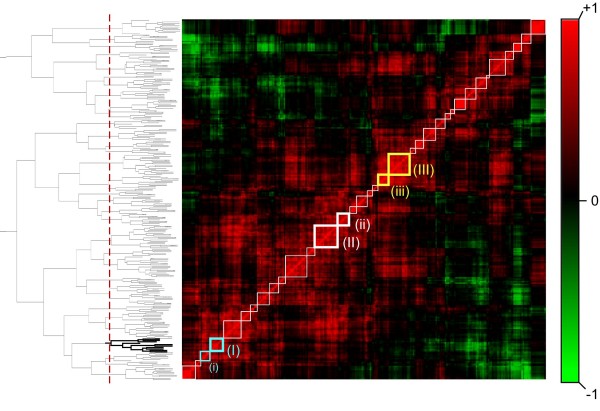
**Identification of gene expression modules in cardiac disease**. Highly expressed genes in the heart were sorted using hierarchical clustering (dendrogram shown on left) based on the similarity in their expression pattern over different types of cardiac disease. These relationships are visualized on a correlation matrix (center), in which each pixel represents the correlation between a pair of genes, and each row represents the relationship between one gene and all others. The color scale (right) represents the degree of correlation, ranging from strongly positive (red) to uncorrelated (black) to strongly negative (green). Boxes along the diagonal of the matrix correspond to modules formed by chopping the dendrogram at the level indicated by the dotted line (determined by maximizing the number of genes in each module associated with the same gene ontology term; see methods). Boxes shown in bold contain genes with particular relevance to cardiac disease, and are shown in detail in figure 3. The bold portion of the dendrogram is also shown in Figure 3.

All highly expressed genes in normal human heart tissue with a mean expression greater than 20,000 units in the Gene Atlas whole-heart data [19] were selected for analysis. This included 298 different Affymetrix probesets monitoring the expression of 222 unique genes. A matrix representing the degree of co-expression between each pair of these probesets was calculated by taking the Pearson correlation coefficient of the cardiomyopathy data table using R [20]. To visualize the matrix of Pearson coefficients for these highly expressed genes, Pearson correlations in this matrix were assigned a color-scale (Figure 2, right) that ranges between red indicating high correlation (R = +1), fading to black indicating no correlation (R = +0), fading to green indicating negative correlation (R = -1).

**Figure 3:**
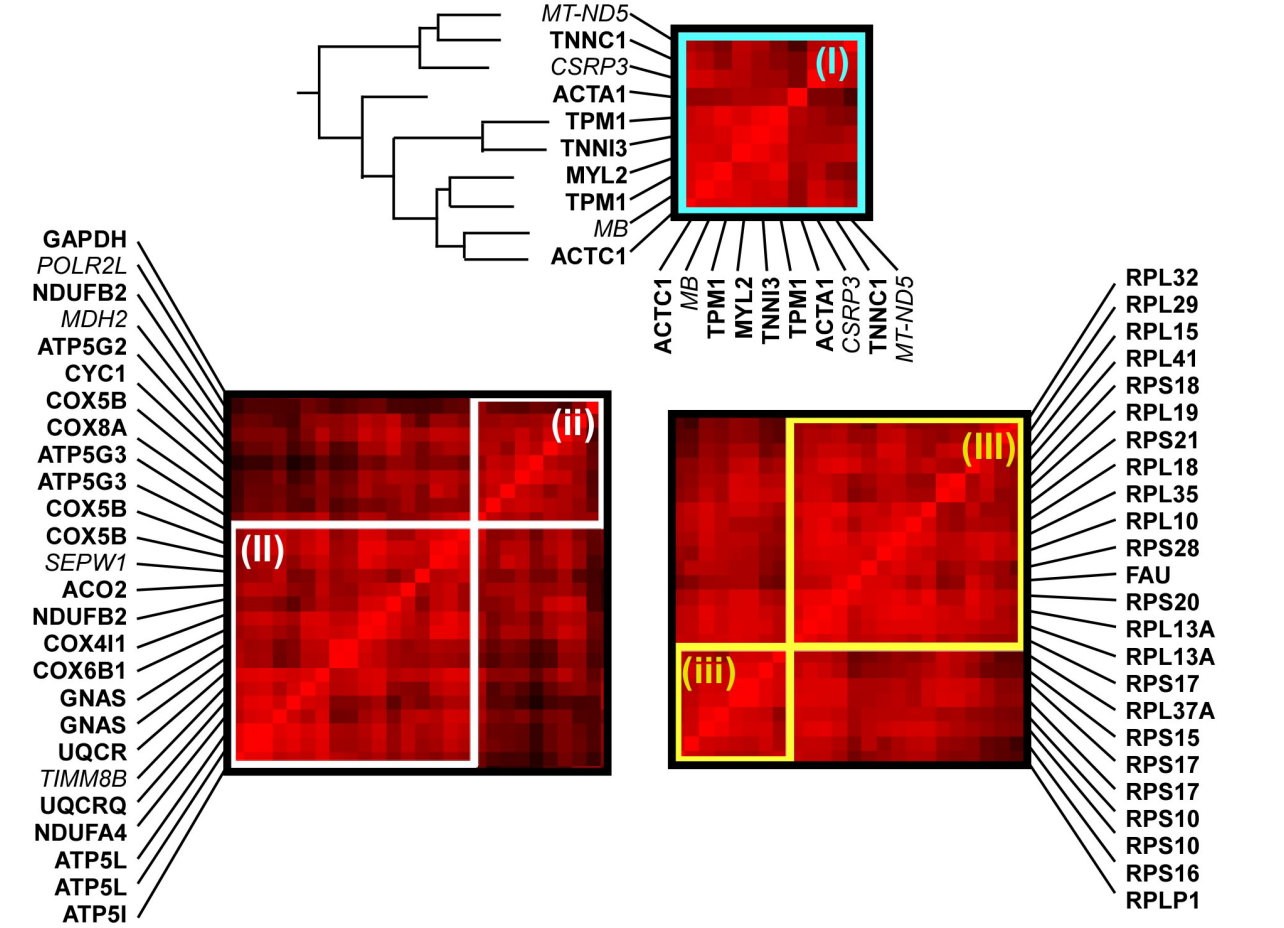
**Detailed composition of gene expression modules identified by the hierarchical clustering algorithm (highlighted boxes in Figure 2)**. Genes in each module are given by MGI ID along the vertical axis. The same gene order is presented along the horizontal axis, as shown for (I). Module (I) is strongly enriched in genes whose products function primarily in the cardiac muscle contractile apparatus (6/9). Note that some genes are represented by two probes (e.g. TPM1) and counted only once. The dendrogram for this module is given on the left (bold section in Figure 2). A heatmap representing the relative expression of each gene over the different cardiac diseases is shown on the right. In the heatmap, color indicates the Z-score of expression relative to the mean over all diseases. Modules (II) and (ii) are enriched in genes involved in generation of precursor metabolites and energy (16/20). Modules (III) and (iii) are entirely comprised of genes involved in protein translation (20/20). Genes whose primary function is not known to match these groups are indicated using italic font.

We sorted this matrix using a procedure based on hierarchical clustering. Clustering was preformed using the R package "Cluster". The data presented here are based on complete-linkage, Euclidean distance hierarchical clustering. Subsequently, gene order was refined using a procedure that maximizes the correlation between neighboring clusters while maintaining the same network of connections in the dendrogram. Briefly, the algorithm proceeds from top to bottom along the dendrogram. At each branch, the mean correlation between neighboring clusters left and right of the new branch on the dendrogram is calculated for both potential orders of the clusters below the branch. The order is set such that it maximizes the mean correlation between neighboring clusters. Since the procedure is applied from top to bottom, it does not affect the composition of clusters, only the order of clusters in the visualization. The resulting visualization is depicted in Figure 2, and details for three clusters are shown in Figure 3.

### Choosing the Optimal Number of Modules that Describe the Data

To determine the optimal number of clusters, we used the hypothesis that genes that are clustered together will share a biological function. Based around this idea, we introduce a measure of uniformity for the biological function of genes in each cluster. This measure can be expressed as: Q_i _= Sum_j_[(N^c^_ij_/N_ij_) * (N_ij_-1)/N^m^_i_]/i, where N^c^_ij _is the number of probesets that share the biological function annotation assigned to cluster j using the minimal Fisher's exact p-value assigned using the Bioconductor package "topGO", N_ij _is the number of probesets in cluster j, N^m^_i _is the mean cluster size (expressed as the number of probesets) when the data is broken into i clusters. The measure was designed such that the contribution of trivial clusters with size 1 is 0, and the contribution from smaller clusters is reduced. The value of this measure for each number of clusters between 2 and 60 is plotted in Additional file 2. At 35 clusters, the measure reaches a global maximum. This number of clusters is used in all subsequent analysis.

### Assembling Promoters for Motif Discovery Analysis

Transcription start sites for each gene selected for detailed promoter analysis were obtained using the database of transcription start sites version 6.0 [21]. When multiple transcription start sites were reported, we chose the most frequent start site reported to be expressed in the heart or pericardium. Promoter sequences from -1 kb to +200 bp were obtained from the ENSEMBL genome browser, release 49, March 2008 [22], as this range allowed us to focus on the strength of IAMMS position filtering algorithm to identify motifs in the core and proximal promoter. A background group of 129 promoter sequences (-1 kb to +200 bp) whose genes show uniform expression across all human tissues was constructed. To build the list of genes, we calculated the ratio of maximum expression to the sum of expression in all tissues across the Gene Atlas. We took the probes with the lowest ratio (indicating uniform expression across tissues). Analysis using DAVID [23] revealed that this group is not enriched in any biological function. For each of these genes, 1 kb of promoter sequence and the entire 5' UTR were obtained using BioMart. The UTR was then truncated at +200 bp to yield the same -1 kb to +200 bp as the foreground set.

### IAMMS De Novo Motif Discovery Search

The upstream region of genes in the contractile, energy generation, and protein translation sets were scanned against the background promoter set using IAMMS, as described previously [24]. In the initial stage, IAMMS detected 10,240, 19,665, and 16,719 motifs to test for enrichment in the contractile, energy, and translation promoter groups, respectively. The p-value of enrichment of each motif in each group was calculated using the hypergeometric distribution, and a p-value threshold of 1e-5 was used to select significantly enriched motifs. At this significance threshold, maximal expected false discovery rates of 0.10, 0.20, and 0.17 were estimated using the Bonferroni method for the contractile, energy generation, and translation promoter sets, respectively.

After motif detection, variations that distinguish contractile, energy, and translation genes were discovered by identifying IUPAC degenerate consensus sequences that optimally separate genes in the each module from those in the other modules. For nearly identical motifs detected independently in energy and translation promoter sequences, occurrences were optimized with respect to genes in the contractile module. To avoid over-fitting, only sequences with a Pearson correlation of 0.9 or above were scanned for enrichment (values of 0.7 were used for Initiator and TATA sequences). Similarity between sequences was measured by concatenating column vectors from the position-weight-matrix representation of a motif, and taking the Pearson correlation between vectors.

### Detection of Known Motifs

To complement the *de novo *search, our next objective was to identify known motifs that are enriched in the promoter. We used the Gene Set Enrichment Analysis (GSEA) software package [25] to identify phylogenetically conserved motifs that are correlated with the contractile, energy generation, or protein translation module. For each module, the 298 highly expressed probesets were ranked based on their correlation to the mean expression patterns of each module. Gene lists were compared against the curated motif gene list ("all", v. 2.5) using GSEA v.2.0.4, requiring motifs to occur in at least 8 of the genes. All motifs that have a false discovery rate corrected q-value less than 0.1 are reported.

### Determining Motif Interval

To determine motif intervals, we built a histogram of the number of occurrences in a sliding window. The mean number of occurrences was calculated based on the size of the motif, assuming a GC content of 50%. We took the interval nearest the transcription start site for which the histogram was above the 99% confidence interval of expected occurrences given a Poisson distribution (as illustrated in Figure 5). Intervals were determined for motifs for which we expected to find a significant bias, either because it was detected by IAMMS as position specific, or (for TATA) because previous literature indicated a significant bias [26].

### Naïve Bayes Classifier

**Figure 4:**
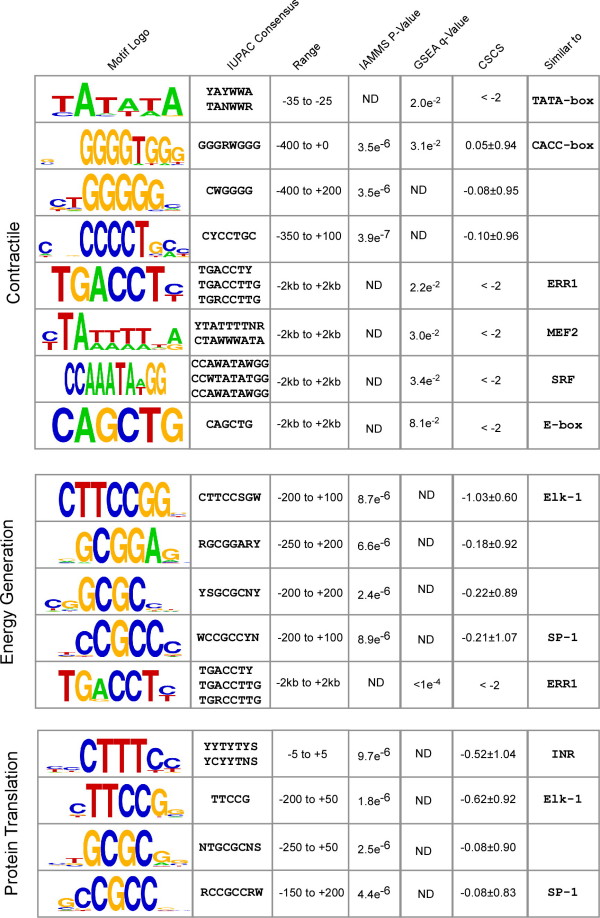
**Top scoring motifs detected in the promoter sequence of genes in the contractile, energy generation, and protein translation modules (Figure 3)**. From the left, columns give: (1) The motif logo; (2) IUPAC consensus sequence(s); (3) Interval relative to the transcription start site in which motif enrichment was observed (+2 to -2 for GSEA matches in which no enrichment detected in the core promoter); (4) P-value of enrichment, identified by IAMMS (ND indicates detection by GSEA); (5) Q-value of enrichment by GSEA (ND indicates detection by IAMMS); (6) Mean phylogenetic conservation score (motifs identified by GSEA are conserved by definition, and thus < -2); (7) Similarity to known motifs. Motifs are ordered based on position relative to the transcription start site or q-value for non-position specific motifs detected by GSEA. IUPAC ambiguity codes are R (A or G), Y (C or T), W (A or T), S (C or G), and N (A, T, C, or G).

The Pearson correlation was calculated between the mean expression patterns for genes in the contractile, energy, and translation module, and all genes designated as "present" in the Gene Atlas heart data [19]. All promoter sequences in the database of transcription start sites [21] corresponding to genes with Pearson correlation above a "high" threshold (0.8, test) or below a "low" threshold (0, background) were obtained for analysis. Other values of "high" and "low" correlation thresholds gave similar results (Additional file 5). The number of occurrences in the respective intervals of each of the motifs in Figure 4 were determined using custom Java software based on the BioJava [27] libraries for position-enriched motifs. GSEA annotations were taken from the file "c3.all.v2.5.symbols.gmt" obtained from the GSEA website, and were applied to each promoter sequence using custom java software. These two sources of annotation were combined into a single data table with columns representing the different IUPAC consensus sequences or GSEA annotations (those shown in Figure 4), and rows representing test and background promoter sequences. Values in this table represented the number of occurrences of each IUPAC consensus sequence in the specified interval in each promoter sequence, or a categorical variable indicating whether the gene is a member of each GSEA gene set.

This data table was used to train and test a naïve Bayes classifier. We use an implementation of the naïve Bayes classifier in the "e1071" R package with default parameters for both training and prediction. The classifier was trained using values based on the promoter sequences of contractile, energy, and translation module depicted in Figure 6 (high co-expression), and 150 of promoters with a Pearson correlation lower than 0 (low co-expression). The classifier was then tested on the remaining promoter sequences not used in training. Default parameters were used for both training and prediction. Statistical analysis of the procedure was completed using Fisher's exact test in R.

**Figure 5:**
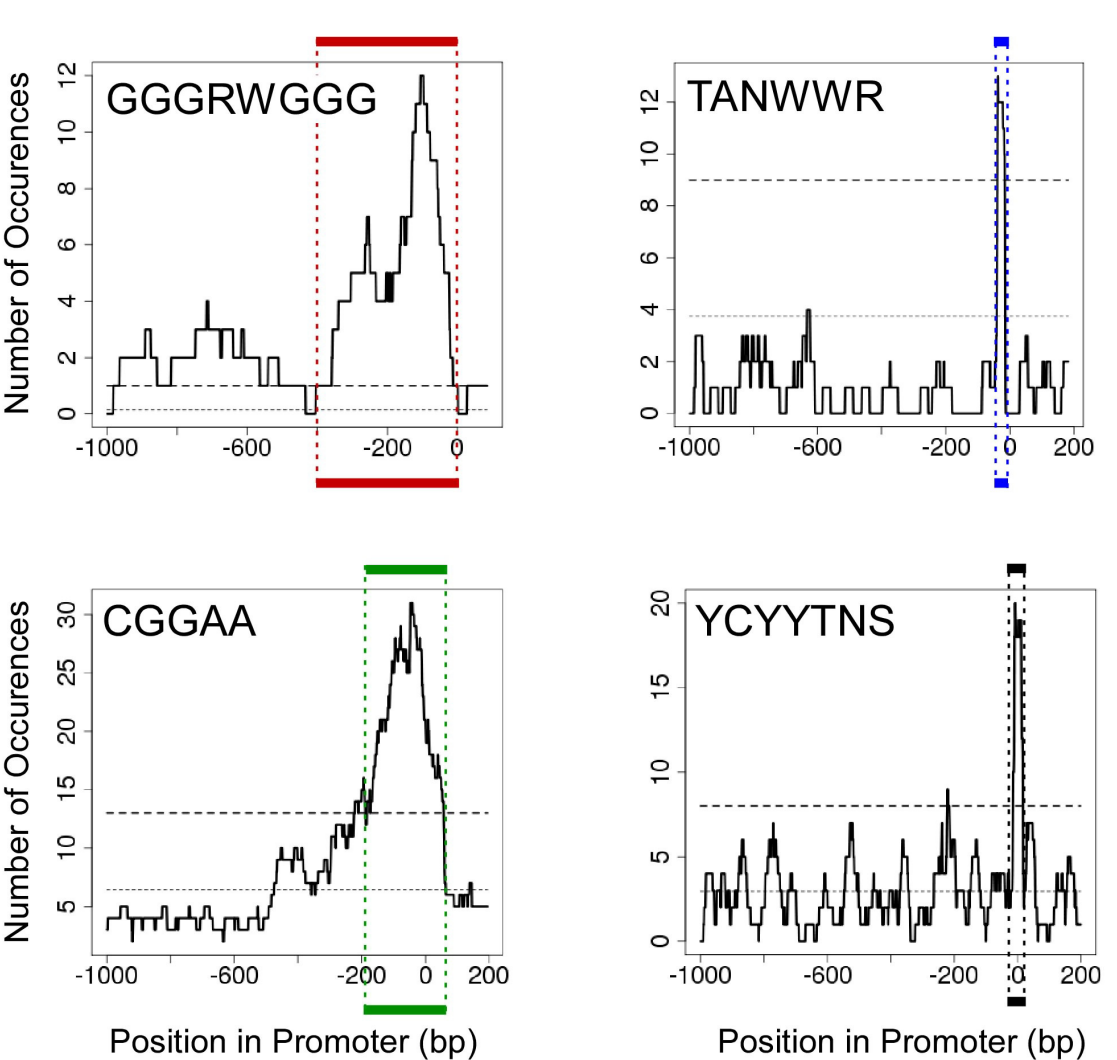
**Identifying the range of motif enrichment**. Each plot tracks the number of occurrences of a given motif in enriched promoter sequences within a sliding window. The lower dotted line indicates the number of occurrences expected by chance. The range in position was determined by taking the approximate point at which the largest peak crosses the 99th percentile (upper dashed line) calculated using a Poisson distribution. For each motif, this range is marked by the colored lines above and below each plot.

**Figure 6:**
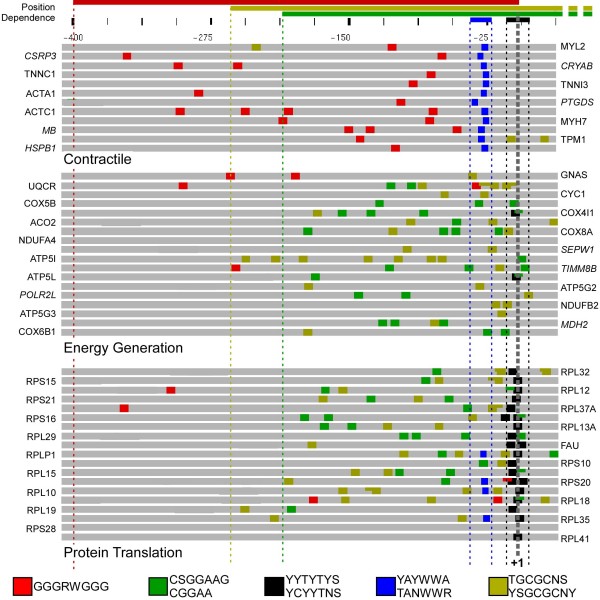
**Specific patterns of top-scoring motifs in the promoter sequence of genes in either the contractile (top), energy generation (center), or protein translation (bottom) modules**. Gray bars represent 435 bp of promoter and 5' UTR sequence (-400 → +35 bp). Colored boxes indicate occurrences of each motif using the color patterns shown in the legend (bottom). Occurrences are drawn to scale, and overlapping sites are drawn at half-height to ease viewing. The scale bar is given on the top, with numbers relative to the transcription start site (gray dashed line, +1 bp). Colored lines above the scale indicate the position range used for each motif. Note that the pyrimidine-rich sequence (black) is directly adjacent to the transcription start site in most genes of the translation module, but not in contractile or energy generation genes. Also note the complete absence of the CCGGAA (green) motif in the contractile promoter sequences. Instead, contractile promoters have a high proportion of a different G-rich motif (GGGRWGGG, red), occurring between -400 bp and the transcription start site.

## Results

### Identifying Gene Expression Modules

Our overall strategy is outlined in Figure 1. The first objective was to identify groups of co-regulated genes (called co-regulatory modules). To this end, we use gene expression microarray data from 149 human ventricle samples collected from public microarray sources, including Gene Expression Omnibus and Harvard's CardioGenomics website (see Methods). Samples were separated into 16 disease conditions (Additional file 1). Different conditions include cardiomyopathies of eight different etiologies, including familial, hypertrophic, idiopathic, ischemic, post-partum, viral, and dilated cardiomyopathy. The response of ischemia and myocardial infarction-induced myocardial failure to treatment by a left ventricular assist device (after LVAD) was also included. Control ("normal") ventricle samples from three different labs were included and treated separately to correct microarray samples for batch effects (see Methods for the normalization procedure).

We started with the identification of co-regulated gene pairs. Figure 2 shows the matrix of pairwise correlation for the 222 highest expressed genes in the Gene Atlas heart data. In our visualization scheme, each pixel represents the degree of co-regulation between a pair of genes. Color represents the square of the Pearson correlation (preserving the sign), with red representing a high Pearson correlation of +1 and green a negative correlation of -1 (see the color scale in Figure 2). Each row (or column) represents the correlation between a single gene and all others. Since the order of genes in the matrix is the same from left to right and bottom to top, the visualization is symmetric about the diagonal. The order of genes is chosen using hierarchical clustering (see Methods), locating co-regulated genes near one another in the matrix.

To identify the most biologically relevant gene expression modules, it was important to identify the best possible divisions between groups of genes provided by the clustering algorithm. We used the hypothesis that the most accurate groups inferred from the data should share a biological function. Therefore, we defined a measure to maximize the number of genes classified as the same biological function in each module (uniformity) and maximize module size (see methods). We found that our measure reaches a global maximum when the data is divided into 35 groups (Additional file 2), indicating that this number is the optimal balance between group size and uniformity of biological function. The divisions indicated by this analysis are denoted on the correlation matrix visualization (Figure 2) by the white, teal, and yellow squares along the diagonal. These divisions are also indicated by the red-dotted line along the dendrogram (Figure 2, left).

Three of the larger modules, enriched in genes with functional relevance to cardiac disease, are shown in detail in Figure 3. In the module shown in Figure 3-I, 6/9 genes (bold font) have a primary function related to muscle contraction (Gene Ontology term GO:0006939). Specific functions include two of three genes in the troponin complex (*TNNI3 *and *TNNC1*), tropomyosin (*TPM1*), cardiac alpha actin (*ACTC1*), and the ventricular myosin light chain isoform (*MYL2*). One additional muscle-specific gene (*CSRP3*) is also likely to play a supporting role in contraction. The gene with the lowest correlation to others in the module, *ACTA1*, is represented by the least-intense red row (r = 0.70, with TNNC1 and CSRP3). This lower correlation is indicative of a relatively large decrease in expression in this gene in one of the dilated cardiomyopathy expression sets (heat maps are shown in Additional file 3). Exceptions to the primary function (names in italics), such as myobglobin (*MB*), are nonetheless strongly co-regulated with other genes in the module (r = 0.83 with *TNNC1*).

In addition to the contractile module, other modules with a primary function related to energy generation and translation are shown in Figure 3. In addition to the large modules shown in Figure 3II and 3III, small neighboring modules enriched for genes with the same function are also shown (Figure 3ii and 3iii). Modules shown in Figure 3II and 3ii contain 16/20 unique genes whose primary function is relevant to the generation of precursor metabolites and energy (GO:0006091). One of the exceptions encodes a selenoprotein (*SEPW1*), an oxygen free-radical scavenger with a role in mitigating the oxidative stress associated with energy generation [28,29]. Modules shown in Figure 3III and 3iii contain 20/20 unique genes that encode proteins in the 80S ribosome complex (GO:0006412). Previous studies have linked both energy generation and protein translation genes with particular types of cardiomyopathy [4,7], highlighting the relevance of these modules to cardiac disease.

### Identification of Putative cis-Elements in the Promoter of Co-regulated Genes

The next step was to identify motifs enriched in these promoter sequences that may be able to explain the observed patterns of co-regulation (Figure 1B). Our hypothesis was that co-regulated genes should share a common set of transcription factor proteins, which could be detected by the presence of a specific pattern of regulatory motifs in the upstream promoter sequence. The three groups highlighted in Figure 3 were chosen for analysis because of their relevance to expression patterns previously observed in cardiac disease [4,7]. We used two strategies to analyze the core promoter region of co-regulated genes. First, the motif discovery algorithm Iterative Alignment/Modular Motif Selection (IAMMS) [24] was used to identify putative regulatory sites *de novo*. Second, we searched for an enrichment of known regulatory motifs using the gene set enrichment analysis (GSEA) tools [25]. To minimize the possibility of false-positive errors, a stringent p-value cutoff threshold (p < 1e-5) was chosen based on a Bonferroni correction for IAMMS, and a corrected q-value cutoff (q < 0.1) for GSEA (see methods). Sequences are presented both as sequence logos and as IUPAC consensus sequences using the degeneracy codes R (A or G), Y (C or T), W (A or T), S (C or G), and N (A, T, C, or G).

The highest scoring motifs enriched in contractile, energy generation, and translation groups are shown in Figure 4. Among the highest scoring motifs enriched in contractile promoter sequences (Figure 4, top) was found to be a degenerate TATA consensus found in all 12 contractile promoter sequences, but only rarely found in energy generation or translation promoters. Several C-rich sequencers were also discovered by IAMMS to be enriched in contractile promoter sequences. One C-rich variation closely resembles a CACC-box known to be involved in the transcriptional regulation of several cardiac genes [30-33]. Our CACC-box includes all of these experimentally validated occurrences, strongly suggesting a functional role for this motif in the other genes as well. Several additional motifs similar to previously characterized cardiac-muscle specific transcription factors were discovered using the GSEA software, including motifs annotated as MEF2 [33], SRF [34], and E-box [35]. GSEA also detected a strong enrichment in a motif annotated as ERR1 (TGACCT), with no previously known role in regulating contractile genes.

Motifs enriched in energy generation and translation promoter sequences are shown in the middle and bottom of Figure 4, respectively. Of the motifs detected as enriched in each set, three nearly identical motifs were detected independently in energy and translation promoters. Similar motifs are GC-rich, including a 4 bp core sequence GCGC, an SP-1-like motif (CCGCC), and a sequence nearly identical to the core of Elk-1 (TTCCGG). Individual motifs that separate energy from translation promoters include a degenerate pyrimidine-rich initiator element enriched near the transcription start site in translation genes (YYCTTTYY), and a GC-rich sequence found to be enriched only in energy generation genes (GCGGA). Energy generation promoters were also found by GSEA to contain the ERR1 motif (TGACCT), which was also discovered independently in the contractile promoter sequences.

For each motif we identified the range of positions in which it is enriched relative to the transcription start site using the approach illustrated in Figure 5. Plots depict the number of occurrences of a given motif found inside a window of fixed size surrounding each point. The top dashed line indicates the 99^th ^percentile of expected occurrences based on a Poisson distribution. We define the range of enrichment to be the interval near the transcription start site in which the number of occurrences of a motif increases above this dashed line, shown by the colored boxes surrounding the plot. The range of any significant bias in distribution discovered using this approach is indicated in Figure 4, and used in subsequent analysis.

Our analysis shows that for each module there is a pattern of regulatory motifs present in nearly all promoter regions that distinguish it from the other modules. Figure 6 shows these patterns plotted to scale in the promoter region of genes in the contractile, energy generation, and protein translation modules. The CACC-box (shown by the red box) occurs in all contractile promoters and only 7/37 energy generation and translation promoter sequences. Similarly, the TATA-like motifs (YAYWWA and TANWWR) can be found in all contractile promoters (dark blue box), but only 4/37 energy generation and translation promoters. Translation promoters are characterized by the presence of a pyrimidine-rich initiator-like element (black box) that appears surrounding the transcription start site in nearly all translation genes (18/19), whereas this motif occurs only twice in energy generation promoters. Occurrences of the Elk-1 motif (green box) and the GC-rich motif (dark yellow box) are in enriched energy generation and translation promoters, but occur rarely in contractile sequences.

We test the hypothesis that the unique pattern of motifs discovered in highly expressed cardiac genes plays a role in the co-regulation of additional genes with similar expression patterns compared to the contractile, energy generation, and protein translation module. Our bioinformatic approach to this question is outlined in Figure 1(C–D). First, we identified additional promoter sequences from across the cardiac transcriptome that were not used in motif discovery, but contain the same pattern of motifs as those in Figure 6 (Figure 1C). Next, we looked at the expression patterns of these additional genes and assessed whether these promoters are more likely to be co-regulated with those used to build the model (Figure 1D). As shown in Table 1, genes with the reported pattern of motifs are significantly more likely to be co-expressed with each module compared with those in the larger population (p = 5.65e-4, 2.03e-3, and 1.16e-11, respectively). The most accurate classifier was based on genes in the Protein Translation module where the odds of having the motif pattern shown in Figure 6 for promoter sequences of highly co-regulated genes (r > 0.8) were 35:51. Conversely, for promoters with low (r < 0) co-expression, the odds of having the motif pattern were 178:1429. The overall strength of association between the presence of the motif pattern and high levels of co-regulation were estimated using the odds ratio OR = (35/51)/(178/1429) = 5.5 (95^th ^Confidence Interval = 3.37 – 8.89), shown at the bottom of Table 1. The classifier based on the contractile and energy promoters also recognized co-regulated genes significantly better than chance, with odds ratios of 2.7 (95^th ^confidence interval = 1.46 – 5.28) and 1.9 (95^th ^confidence interval = 1.24 – 3.13), respectively. Moreover, the statistical significance of each classifier is robust across a wide range of definitions for high and low co-regulation (Additional file 5). This provides strong evidence that the pattern of motifs described here plays a role in the changes in gene expression observed in heart disease.

**Table 1:** The pattern of motifs in the core promoter has a significant association with gene expression in a group of holdout genes not used in motif discovery analysis.

		Module					
Correlation (microarray):		Contractile		Energy Generation		Protein Translation	
		High	Low	High	Low	High	Low
Pattern:	Yes:	74	1638	152	1371	35	178
	No:	13	768	25	437	51	1429

Odds Ratio:		**2.66 **(1.46 – 5.28)		**1.93 **(1.24 – 3.13)		**5.50 **(3.37 – 8.89)	
Fisher's p <		5.65e^-4^		2.03e^-3^		1.16e^-11^	

Contingency tables show the relationship between the pattern of motifs in the core promoter region and the expression patterns of genes in the test group (n = 3473), not used during motif the discovery procedure. The correlation thresholds were set to r > 0.8 (high) and r < 0 (low). The odds ratio measures the strength of association between the pattern of motifs in each promoter and the pattern of expression. The 95% confidence interval of the odds ratio is shown in parentheses. The p-value calculated using Fisher's exact test is given in the bottom row.

## Discussion

In the present study, we divide genes into groups, or modules, that undergo similar changes in their expression patterns over different forms of heart failure. The modules indicated by our analysis are exploited to identify putative *cis*-regulatory motifs which may bind transcription factors that contribute to the observed pathological expression patterns. Our approach was motivated by the hypothesis that genes with similar expression patterns are regulated by the same set of transcription factors, and therefore are likely to have similar *cis*-regulatory motifs in their upstream promoter region. The power of this assumption was recently demonstrated in the yeast model [15], where motifs were discovered that were able to predict gene expression patterns. To our knowledge, this article is the first in which this assumption has been applied to human tissue or to the study of a specific disease in which a holdout set of genes has been used to validate predictions. We report 17 putative *cis*-regulatory motifs, including ELK-1, a CACC-box, and a pyrimidine-rich initiatory variant that are predicted to play a role in cardiac gene expression. Our predictions may serve as a base for experimental studies seeking to understand how genes are reorganized during heart failure.

We discovered several motifs that are similar to known regulatory elements. Of these, many are variations of either core promoter (TATA, Initiator) or proximal enhancer (ELK-1, CACC, SP-1) elements. We show here that these motifs are more likely to be found together in promoter sequences that drive specific changes in gene expression during heart disease (Table 1). This implies that the motifs discovered here are strong candidates for modulating disease-related changes in gene expression. Our study highlights the importance of core and proximal promoter motifs in determining changes in transcription, and suggest that they play a larger role in the combinational regulatory code than has previously been ascribed.

Promoter regions of genes in the contractile module are characterized by the presence of a TATA variation (TANWWR), CACC box (GGGRWGG), MEF2 (YTAWWWWWTR), SRF (CCWWWWWWGG), and an E-box (CAGCTG). Single-promoter experimental studies have associated both all of these motifs with certain contractile promoters [30-37]. For instance, an AT-rich sequence that resembles a TATA-box has been previously identified in the promoter of *TNNI3 *[30], *TNNC1 *[36], *MYH7 *[37], and MB [33]. Similarly, the CACC box has also been previously identified in certain contractile-related promoter sequences, including *TNNI3 *[30,31], *TNNC1 *[32], and *MB *[33]. All of these experimentally verified occurrences were discovered by our motif discovery strategy, along with previously unknown occurrences in nine additional promoter sequences, including *ACTA1*, *MYL2*, *TNNC1*, *MYH7*, *TPM1*, *HSPB1*, *CRYAB, CSRP3*, and *PTGDS*. Our results complement the single-promoter experimental studies, and suggest that many of these motifs play a larger role in mediating cardiac gene expression than previously anticipated.

At the level of the core promoter, energy generation and protein translation genes are controlled by surprisingly similar regulatory programs. Of four motifs discovered as significantly enriched in both energy and translation sequences, three share nearly identical GC-rich core sequences (Figure 4), including an Elk-1 binding site (CCGGAA), a motif with a GC-rich core sequence (GCGC), and a degenerate SP-1 like motif (CCGCC). These motifs were all found to be enriched in additional promoter sequences not used in the motif discovery procedure that share the expression patterns with the energy or translation module (not shown; p < 0.02) and were important for our naïve Bayes classification analysis. These results suggest that these factors may play a role in mediating gene expression changes during cardiac disease.

The Elk-1 prediction has particular relevance to the study of cardiac disease, because Elk-1 activity is modulated by both MAPK-p38 and calcineurin signaling pathways [38] that are implicated in animal models of heart failure (Reviewed in [39]). Calcineurin signaling is activated by calcium, particularly under conditions of calcium overload that are a nearly universal effect of end-stage heart failure. The literature on MAPK-p38 in heart failure suggests that p38 is activated, at least in the early stages of animal models of hypertrophy [40]. Unfortunately, the two signaling pathways have opposite effects on Elk-1 activity [41,38], making it difficult to predict how a particular heart disease will affect the transcription of Elk-1 targets. Experimental studies in human patients have directly demonstrated changes in Elk-1 signaling during heart failure [42], however, highlighting the importance of discovering heart-specific targets. Our analysis suggests that Elk-1 occurrences bind preferentially in the promoters of energy and translation genes and may be relevant to understanding changes in expression during cardiac disease.

We also discovered a novel motif with a GC-rich core sequence (GCGC) that occurs many times in the promoter of genes in the energy and translation modules. In addition to this motif, we also found a longer, non-position specific motif highly enriched in the same promoter sequences that closely resembles two nearby position-specific GC-rich half sites (Additional file 4). Moreover, we find that at least seven promoters contain either directly adjacent or partially overlapping occurrences of the short GCGC-rich sequence (dark yellow motif, shown in Figure 6), which is consistent with the idea that this finding represents half of a longer motif. Occurrences of the longer motif are highly conserved through evolution compared to surrounding sequence (Additional file 4), strongly suggesting that this motif plays a functional role.

A non-canonical initiator motif (YYCTTTYY) appears to be a nearly universal feature of translation promoter sequences. In addition to its sequence, the motif is highly specific to the positive strand and the area immediately surrounding the transcription start site (Figure 5, bottom right panel). Given the pyrimidine-rich sequence and the unambiguous bias in position, it is clear that this motif is a degenerate initiator consensus common to the promoter of translation genes. A similar motif has been identified in a previous study [43] and found to be enriched in translation promoter sequences. Here, we identified this motif in the promoter of a group of co-regulated genes, suggesting that it may play a direct role in transcriptional regulation. Given that the initiator consensus is the initial binding site for RNA polymerase II, it is not far-fetched to speculate that small differences in the sequence may play a role in determining either the affinity of polymerase II or the energy required to separate the DNA strands and initiate transcription; both of which could potentially modulate the quantity of transcript produced. Nonetheless, we cannot rule out the possibility that this motif it is just more likely to occur in combination with another motif (such as Elk-1) that more directly determines the level of transcription.

## Conclusion

We describe an analysis of public microarray experiments that identifies groups of genes, or co-regulatory modules, that undergo similar changes in expression over different forms of hypertrophic and dilated cardiomyopathy. Three of these modules were associated with cardiac disease by previous microarray studies. The promoter sequence of genes in these modules were used to identify putative regulatory motifs predicted to cause the observed changes in gene expression. Our analysis discovered 17 regulatory motifs, including a CACC-box, Elk-1, a degenerate initiator sequence, and a novel GC-rich motif. Searching for the reported pattern of motifs in additional promoter sequences (not used for motif discovery) reveals that promoters with each pattern are significantly more likely to drive similar changes in gene expression in the cardiomyopathies analyzed in this study. Our analysis reveals motifs predicted to play a role in gene expression changes associated with several different types of human heart disease.

## Abbreviations

IAMMS: Iterative Alignment/Modular Motif Selection; GO: Gene ontology; GSEA: Gene Set Enrichment Analysis; LVAD: Left ventricular assist device; MI: Myocardial infarction.

## Competing interests

The authors declare that they have no competing interests.

## Authors' contributions

CGD contributed to the study design, wrote all software and script files, conducted the analysis, prepared the figures, and drafted the manuscript. AMP assisted in study design and manuscript preparation. All authors read and approved the final manuscript.

## Pre-publication history

The pre-publication history for this paper can be accessed here:

http://www.biomedcentral.com/1755-8794/2/31/prepub

## Supplementary Material

Additional file 1 **Sources of human ventricular microarray data collected for the present study**. Sources of human ventricular microarray data collected for the present study. Samples were collected from four different experimental data sources, for which the Gene Expression Omnibus experiment ID is given (column 1). Sample sources include 16 physiological conditions separated by study and disease state (column 2). Collectively, this data represents 149 different microarray samples (column 3). The last column gives references for the original publication (column 4). Abbreviations: *LVAD – left ventricular assist device; **MI – myocardial infarction. Click here for file

Additional file 2 **Plots of the uniformity score, used to determine the optimal number of clusters**. The determination of co-regulatory modules by plotting the uniformity score against the number of clusters. The uniformity score optimizes the proportion of genes that share a biological function with respect to the cluster size (see methods). The uniformity score reaches a maximum at 35 clusters, shown expanded in the insert. Click here for file

Additional file 3 **Heatmap of genes in the contractile, energy, and translation module over different cardiomyopathies**. Heatmap of genes in the myocardial contraction (A), energy generation (B), and protein translation (C) module over the different cardiomyopathies examined in the present study. Heatmaps are presented side-by-side with correlation visualizations from the text to ease comparison. Color indicates the Z-score of expression relative to the mean over all diseases. The order of genes is the same as presented in the text. Note that the order in which cardiomyopathies are presented are not the same among the different heatmaps. Click here for file

Additional file 4 **A *cis*-element enriched in promoters driving expression of genes in the energy generation module**. A sample *cis*-element enriched in promoters driving the expression of genes in the energy generation module. (A) The motif logo. (B) The cross-promoter alignment. Columns, from left to right, give the MGI symbol, start position, strand relative to the transcription start site (+1), consensus sequence (shown on top), and cross-species conservation score (CSCS; negative indicates strong phylogenetic conservation). (C-D) Sample phylogenetic alignments for occurrences in ATP5G3 (CSCS = -1.0, C) and ATP5L (CSCS = -1.9, D). Click here for file

Additional file 5 **Plot of negative log of p-value as a function of high and low correlation cutoff thresholds**. Negative log of p-values represent the enrichment of the pattern of motifs in promoter sequences that drive highly correlated ("high") expression with respect to uncorrelated ("low") expression. Values along the horizontal axis are the Pearson correlation above which genes are classified as "highly" correlated (between 0.65 and 0.85; top), or below which genes are classified as having a "low" correlation (bottom; -0.10 to 0.10), as described in Methods. P-values are plotted separately for classification based on the contractile (black), energy generation (green) and protein translation (red) module. High correlation thresholds (top) are plotted with respect to a constant low correlation threshold (R = 0.0). Low correlation thresholds (bottom) are plotted using a constant high correlation threshold (R = 0.80). The dotted line represents a p-value cutoff of 0.05, indicating that 23 of 24 parameter combinations are statistically significant at this cutoff threshold (p < 0.05). This plot demonstrates that the results of the naïve Bayes classification are robust to changes in these parameters. Click here for file

Additional file 6 **Genes comprising each of the 35 clusters chosen for analysis in the article**. The cluster number, Affymetrix ID, and MGI symbol (if available) of each of the 271 genes used in the analysis zipped into a tab delimited and Microsoft Excel file format. Click here for file

## References

[B1] Towbin JA Bowles NE Molecular genetics of left ventricular dysfunction Curr Mol Med 2001 1 81 90 11899244 10.2174/1566524013364077

[B2] Barrans JD Allen PD Stamatiou D Dzau VJ Liew C Global Gene Expression Profiling of End-Stage Dilated Cardiomyopathy Using a Human Cardiovascular-Based cDNA Microarray Am J Pathol 2002 160 2035 2043 12057908 10.1016/S0002-9440(10)61153-4 PMC1850841

[B3] Wittchen F Suckau L Witt H Skurk C Lassner D Fechner H Sipo I Ungethüm U Ruiz P Pauschinger M Tschope C Rauch U Kühl U Schultheiss H Poller W Genomic expression profiling of human inflammatory cardiomyopathy (DCMi) suggests novel therapeutic targets Journal of Molecular Medicine (Berlin, Germany) 2007 85 257 271 17106732 10.1007/s00109-006-0122-9 PMC1820750

[B4] Hwang J Allen PD Tseng GC Lam C Fananapazir L Dzau VJ Liew C Microarray gene expression profiles in dilated and hypertrophic cardiomyopathic end-stage heart failure Physiol Genomics 2002 10 31 44 12118103 10.1152/physiolgenomics.00122.2001

[B5] Kittleson MM Ye SQ Irizarry RA Minhas KM Edness G Conte JV Parmigiani G Miller LW Chen Y Hall JL Garcia JG Hare JM Identification of a Gene Expression Profile That Differentiates Between Ischemic and Nonischemic Cardiomyopathy Circulation 2004 110 3444 3451 15557369 10.1161/01.CIR.0000148178.19465.11

[B6] Tan F Moravec CS Li J Apperson-Hansen C McCarthy PM Young JB Bond M The gene expression fingerprint of human heart failure Proc Natl Acad Sci USA 2002 99 11387 92 12177426 10.1073/pnas.162370099 PMC123266

[B7] Grzeskowiak R Witt H Drungowski M Thermann R Hennig S Perrot A Osterziel KJ Klingbiel D Scheid S Spang R Lehrach H Ruiz P Expression profiling of human idiopathic dilated cardiomyopathy Cardiovasc Res 2003 59 400 411 12909323 10.1016/s0008-6363(03)00426-7

[B8] Hall JL Grindle S Han X Fermin D Park S Chen Y Bache RJ Mariash A Guan Z Ormaza S Thompson J Graziano J de Sam Lazaro SE Pan S Simari RD Miller LW Genomic profiling of the human heart before and after mechanical support with a ventricular assist device reveals alterations in vascular signaling networks Physiol Genomics 2004 17 283 291 14872006 10.1152/physiolgenomics.00004.2004

[B9] Barth AS Kuner R Buness A Ruschhaupt M Merk S Zwermann L Kääb S Kreuzer E Steinbeck G Mansmann U Poustka A Nabauer M Sültmann H Identification of a Common Gene Expression Signature in Dilated Cardiomyopathy Across Independent Microarray Studies Journal of the American College of Cardiology 2006 48 1610 1617 17045896 10.1016/j.jacc.2006.07.026

[B10] Eisen MB Spellman PT Brown PO Botstein D Cluster analysis and display of genome-wide expression patterns Proc Natl Acad Sci USA 1998 95 14863 8 9843981 10.1073/pnas.95.25.14863 PMC24541

[B11] Iyer VR Eisen MB Ross DT Schuler G Moore T Lee JC Trent JM Staudt LM Hudson J Boguski MS Lashkari D Shalon D Botstein D Brown PO The Transcriptional Program in the Response of Human Fibroblasts to Serum Science 1999 283 83 87 9872747 10.1126/science.283.5398.83

[B12] Segal E Shapira M Regev A Pe'er D Botstein D Koller D Friedman N Module networks: identifying regulatory modules and their condition-specific regulators from gene expression data Nat Genet 2003 34 166 176 12740579 10.1038/ng1165

[B13] Gupta M Liu JS De novo cis-regulatory module elicitation for eukaryotic genomes Proc Natl Acad Sci USA 2005 102 7079 7084 15883375 10.1073/pnas.0408743102 PMC1129096

[B14] Kel-Margoulis OV Romashchenko AG Kolchanov NA Wingender E Kel AE COMPEL: a database on composite regulatory elements providing combinatorial transcriptional regulation Nucl Acids Res 2000 28 311 315 10592258 10.1093/nar/28.1.311 PMC102399

[B15] Beer MA Tavazoie S Predicting Gene Expression from Sequence Cell 2004 117 185 198 15084257 10.1016/s0092-8674(04)00304-6

[B16] Edgar R Domrachev M Lash AE Gene Expression Omnibus: NCBI gene expression and hybridization array data repository Nucleic Acids Res 2002 30 207 210 11752295 10.1093/nar/30.1.207 PMC99122

[B17] Gentleman R Carey V Bates D Bolstad B Dettling M Dudoit S Ellis B Gautier L Ge Y Gentry J Hornik K Hothorn T Huber W Iacus S Irizarry R Leisch F Li C Maechler M Rossini A Sawitzki G Smith C Smyth G Tierney L Yang J Zhang J Bioconductor: open software development for computational biology and bioinformatics Genome Biology 2004 5 15461798 10.1186/gb-2004-5-10-r80 PMC545600

[B18] Johnson WE Li C Rabinovic A Adjusting batch effects in microarray expression data using empirical Bayes methods Biostatistics 2007 8 118 127 16632515 10.1093/biostatistics/kxj037

[B19] Su AI Wiltshire T Batalov S Lapp H Ching KA Block D Zhang J Soden R Hayakawa M Kreiman G Cooke MP Walker JR Hogenesch JB A gene atlas of the mouse and human protein-encoding transcriptomes Proc Natl Acad Sci USA 2004 101 6062 6067 15075390 10.1073/pnas.0400782101 PMC395923

[B20] R Development Core Team R: A Language and Environment for Statistical Computing 2008 http://www.R-project.org

[B21] Wakaguri H Yamashita R Suzuki Y Sugano S Nakai K DBTSS: database of transcription start sites, progress report 2008 Nucleic Acids Res 2008 36 D97 101 17942421 10.1093/nar/gkm901 PMC2238895

[B22] Hubbard TJP Aken BL Beal K Ballester B Caccamo M Chen Y Clarke L Coates G Cunningham F Cutts T Down T Dyer SC Fitzgerald S Fernandez-Banet J Graf S Haider S Hammond M Herrero J Holland R Howe K Howe K Johnson N Kahari A Keefe D Kokocinski F Kulesha E Lawson D Longden I Melsopp C Megy K Ensembl 2007 Nucl Acids Res 2007 D610 7 17148474 10.1093/nar/gkl996 PMC1761443

[B23] Dennis G Sherman BT Hosack DA Yang J Gao W Lane HC Lempicki RA DAVID: Database for Annotation, Visualization, and Integrated Discovery Genome Biol 2003 4 PMC193660 12734009

[B24] Danko C McIlvain V Qin M Knox B Pertsov A Bioinformatic identification of novel putative photoreceptor specific cis-elements BMC Bioinformatics 2007 8 407 17953763 10.1186/1471-2105-8-407 PMC2225425

[B25] Subramanian A Tamayo P Mootha VK Mukherjee S Ebert BL Gillette MA Paulovich A Pomeroy SL Golub TR Lander ES Mesirov JP Gene set enrichment analysis: a knowledge-based approach for interpreting genome-wide expression profiles Proc Natl Acad Sci USA 2005 102 15545 15550 16199517 10.1073/pnas.0506580102 PMC1239896

[B26] Xie X Lu J Kulbokas EJ Golub TR Mootha V Lindblad-Toh K Lander ES Kellis M Systematic discovery of regulatory motifs in human promoters and 3[prime] UTRs by comparison of several mammals Nature 2005 434 338 345 15735639 10.1038/nature03441 PMC2923337

[B27] Holland RCG Down TA Pocock M Prlic A Huen D James K Foisy S Drager A Yates A Heuer M Schreiber MJ BioJava: an open-source framework for bioinformatics Bioinformatics 2008 24 2096 2097 18689808 10.1093/bioinformatics/btn397 PMC2530884

[B28] Jeong DW Kim TS Chung YW Lee BJ Kim IY Selenoprotein W is a glutathione-dependent antioxidant in vivo FEBS Lett 2002 517 225 228 12062442 10.1016/s0014-5793(02)02628-5

[B29] Kioussi C Whanger P Selenoprotein W in development and oxidative stress Selenium 2006 135 140 10.1016/j.jinorgbio.2006.05.018 16876868

[B30] Bhavsar PK Dellow KA Yacoub MH Brand NJ Barton PJ Identification of cis-acting DNA elements required for expression of the human cardiac troponin I gene promoter J Mol Cell Cardiol 2000 32 95 108 10652194 10.1006/jmcc.1999.1058

[B31] Dellow KA Bhavsar PK Brand NJ Barton PJ Identification of novel, cardiac-restricted transcription factors binding to a CACC-box within the human cardiac troponin I promoter Cardiovasc Res 2001 50 24 33 11282075 10.1016/s0008-6363(01)00204-8

[B32] Parmacek MS Vora AJ Shen T Barr E Jung F Leiden JM Identification and characterization of a cardiac-specific transcriptional regulatory element in the slow/cardiac troponin C gene Mol Cell Biol 1992 12 1967 1976 1569934 10.1128/mcb.12.5.1967-1976.1992 PMC364367

[B33] Bassel-Duby R Grohe CM Jessen ME Parsons WJ Richardson JA Chao R Grayson J Ring WS Williams RS Sequence elements required for transcriptional activity of the human myoglobin promoter in intact myocardium Circ Res 1993 73 360 368 8330378 10.1161/01.res.73.2.360

[B34] Parmacek MS Myocardin-Related Transcription Factors: Critical Coactivators Regulating Cardiovascular Development and Adaptation Circ Res 2007 100 633 644 17363709 10.1161/01.RES.0000259563.61091.e8

[B35] Malik S Huang CF Schmidt J The role of the CANNTG promoter element (E box) and the myocyte-enhancer-binding-factor-2 (MEF-2) site in the transcriptional regulation of the chick myogenin gene Eur J Biochem 1995 230 88 96 7601128 10.1111/j.1432-1033.1995.tb20537.x

[B36] Christensen TH Prentice H Gahlmann R Kedes L Regulation of the human cardiac/slow-twitch troponin C gene by multiple, cooperative, cell-type-specific, and MyoD-responsive elements Mol Cell Biol 1993 13 6752 6765 8413270 10.1128/mcb.13.11.6752-6765.1993 PMC364738

[B37] Rindt H Gulick J Knotts S Neumann J Robbins J In vivo analysis of the murine beta-myosin heavy chain gene promoter J Biol Chem 1993 268 5332 5338 8444907

[B38] Tian J Karin M Stimulation of Elk1 Transcriptional Activity by Mitogen-activated Protein Kinases Is Negatively Regulated by Protein Phosphatase 2B (Calcineurin) J Biol Chem 1999 274 15173 15180 10329725 10.1074/jbc.274.21.15173

[B39] Wilkins BJ Molkentin JD Calcineurin and cardiac hypertrophy: where have we been? Where are we going? J Physiol 2002 541 1 8 12015416 10.1113/jphysiol.2002.017129 PMC2290305

[B40] Megeney LA Kablar B Perry RL Ying C May L Rudnicki MA Severe cardiomyopathy in mice lacking dystrophin and MyoD Proc Natl Acad Sci USA 1999 96 220 225 9874799 10.1073/pnas.96.1.220 PMC15120

[B41] Sugimoto T Stewart S Guan KL The calcium/calmodulin-dependent protein phosphatase calcineurin is the major Elk-1 phosphatase J Biol Chem 1997 272 29415 29418 9367995 10.1074/jbc.272.47.29415

[B42] Flesch M Margulies KB Mochmann H Engel D Sivasubramanian N Mann DL Differential Regulation of Mitogen-Activated Protein Kinases in the Failing Human Heart in Response to Mechanical Unloading Circulation 2001 104 2273 2276 11696464 10.1161/hc4401.099449

[B43] Wang J Hannenhalli S A mammalian promoter model links cis elements to genetic networks Biochem Biophys Res Commun 2006 347 166 177 16806065 10.1016/j.bbrc.2006.06.062

