# Supplementary figures and images for: Identification of gene co-regulatory modules and associated cis-elements involved in degenerative heart disease
Source: BMC Med Genomics. 2009 May 28;2:31. doi: 10.1186/1755-8794-2-31 (PMC2700136; doi:10.1186/1755-8794-2-31)

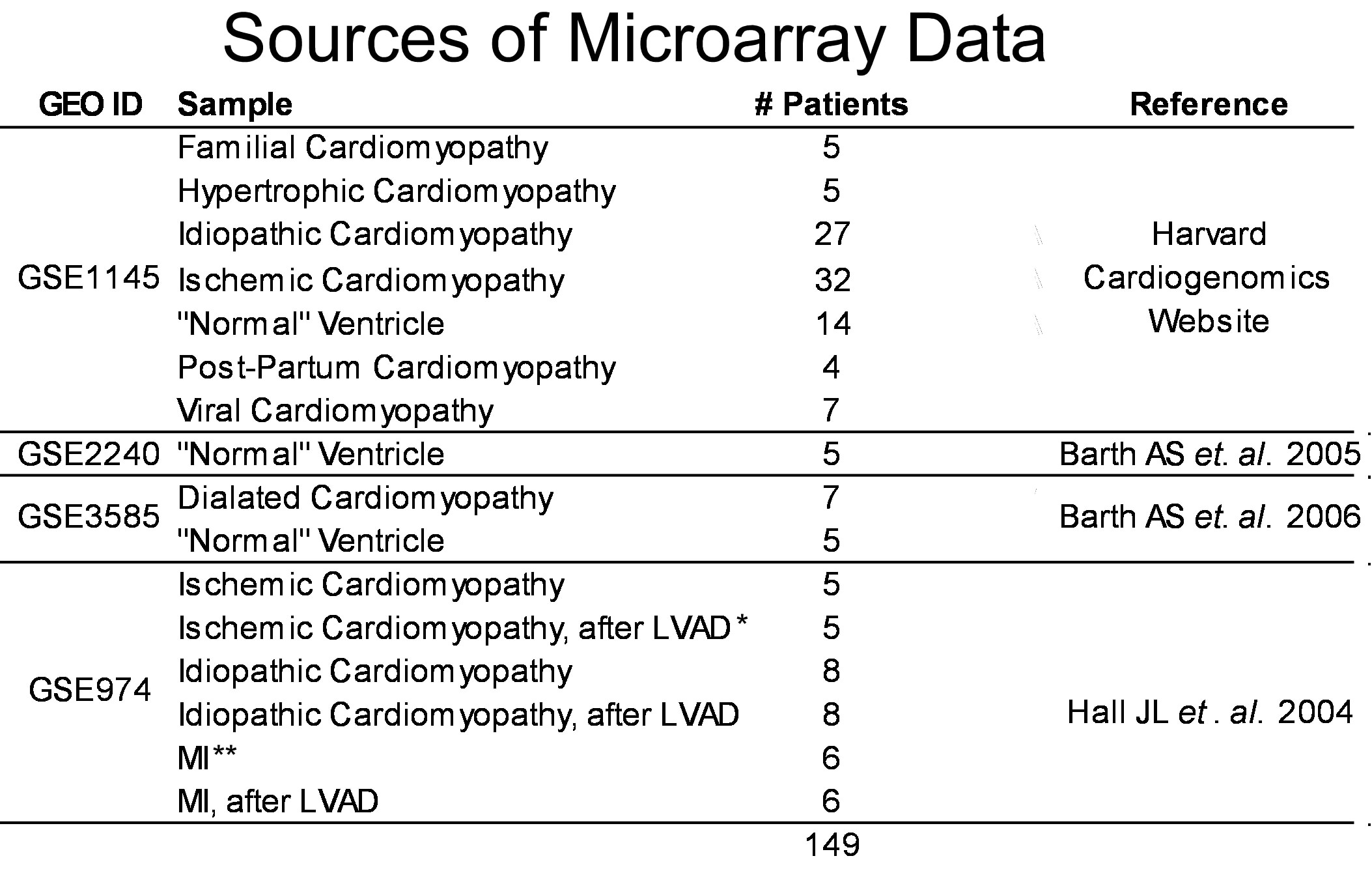

Supplement: Additional file 1 — Sources of human ventricular microarray data collected for the present study. Sources of human ventricular microarray data collected for the present study. Samples were collected from four different experimental data sources, for which the Gene Expression Omnibus experiment ID is given (column 1). Sample sources include 16 physiological conditions separated by study and disease state (column 2). Collectively, this data represents 149 different microarray samples (column 3). The last column gives references for the original publication (column 4). Abbreviations: *LVAD – left ventricular assist device; **MI – myocardial infarction. [file 1755-8794-2-31-S1.jpeg]

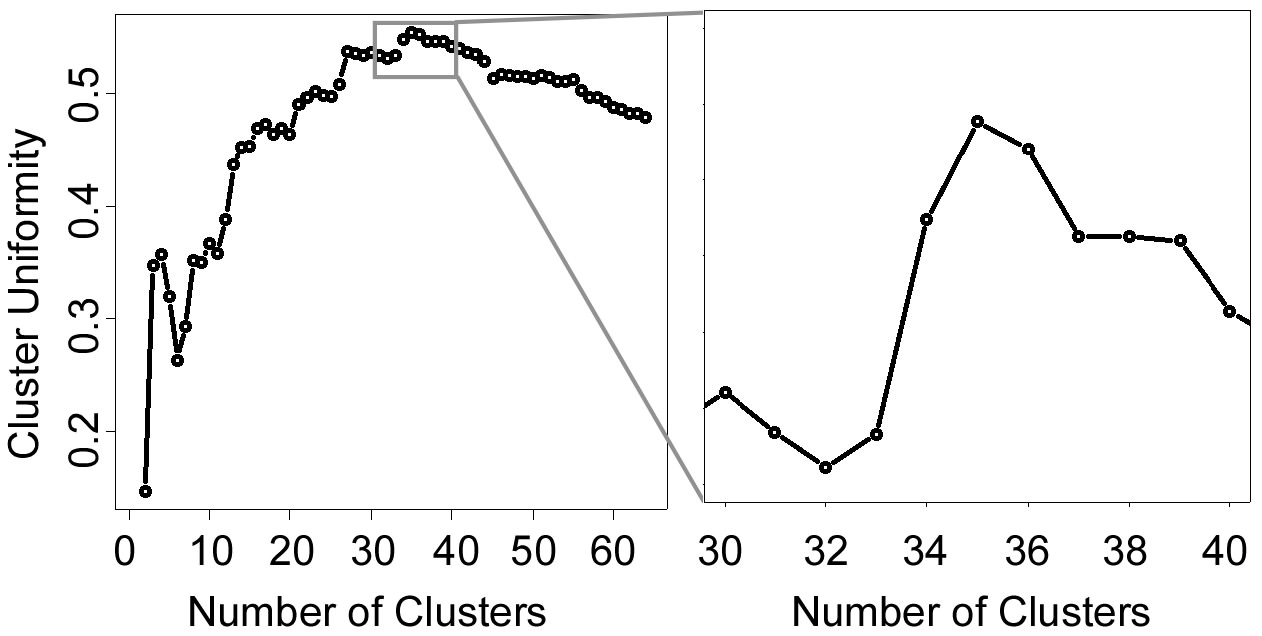

Supplement: Additional file 2 — Plots of the uniformity score, used to determine the optimal number of clusters. The determination of co-regulatory modules by plotting the uniformity score against the number of clusters. The uniformity score optimizes the proportion of genes that share a biological function with respect to the cluster size (see methods). The uniformity score reaches a maximum at 35 clusters, shown expanded in the insert. [file 1755-8794-2-31-S2.jpeg]

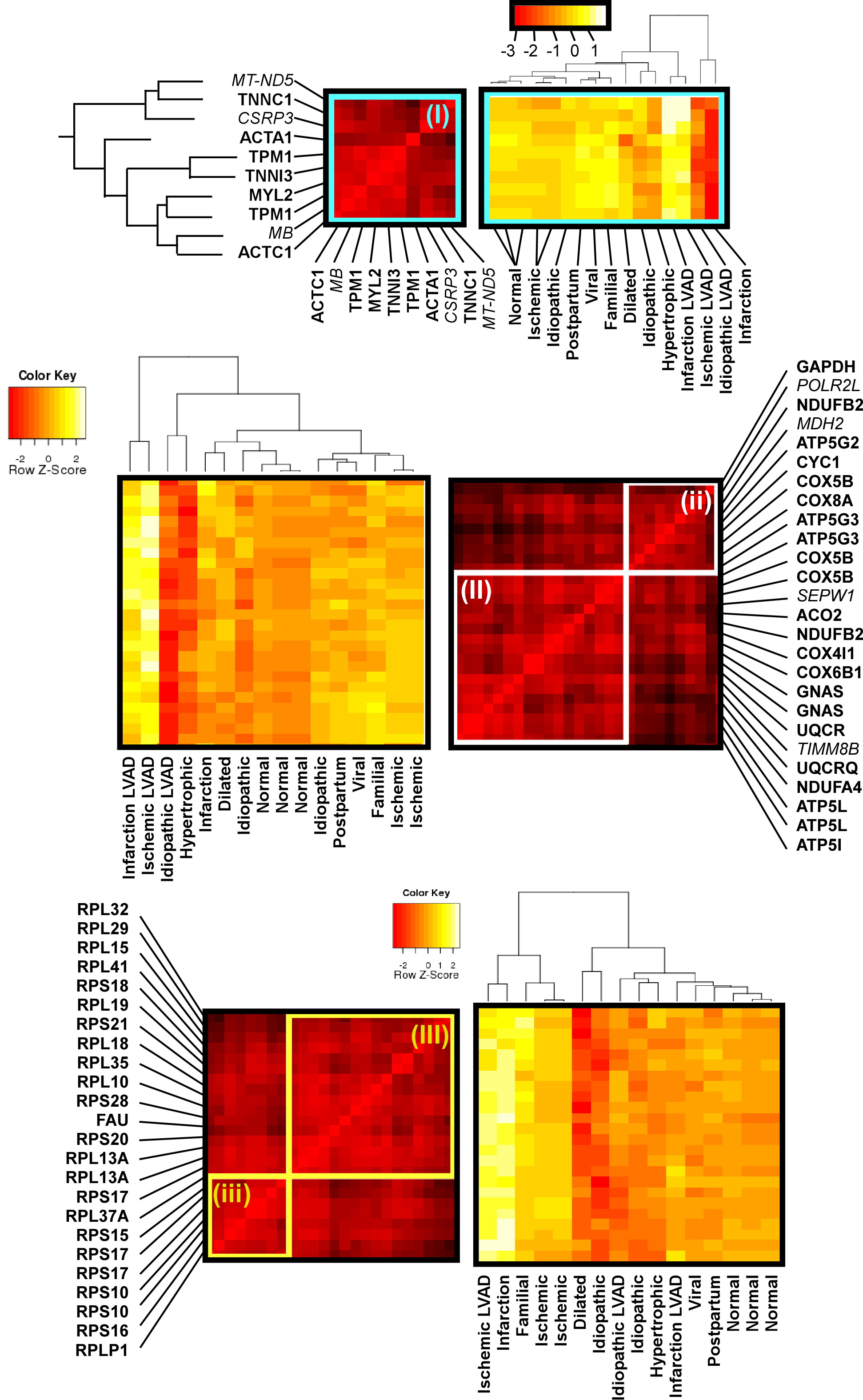

Supplement: Additional file 3 — Heatmap of genes in the contractile, energy, and translation module over different cardiomyopathies. Heatmap of genes in the myocardial contraction (A), energy generation (B), and protein translation (C) module over the different cardiomyopathies examined in the present study. Heatmaps are presented side-by-side with correlation visualizations from the text to ease comparison. Color indicates the Z-score of expression relative to the mean over all diseases. The order of genes is the same as presented in the text. Note that the order in which cardiomyopathies are presented are not the same among the different heatmaps. [file 1755-8794-2-31-S3.jpeg]

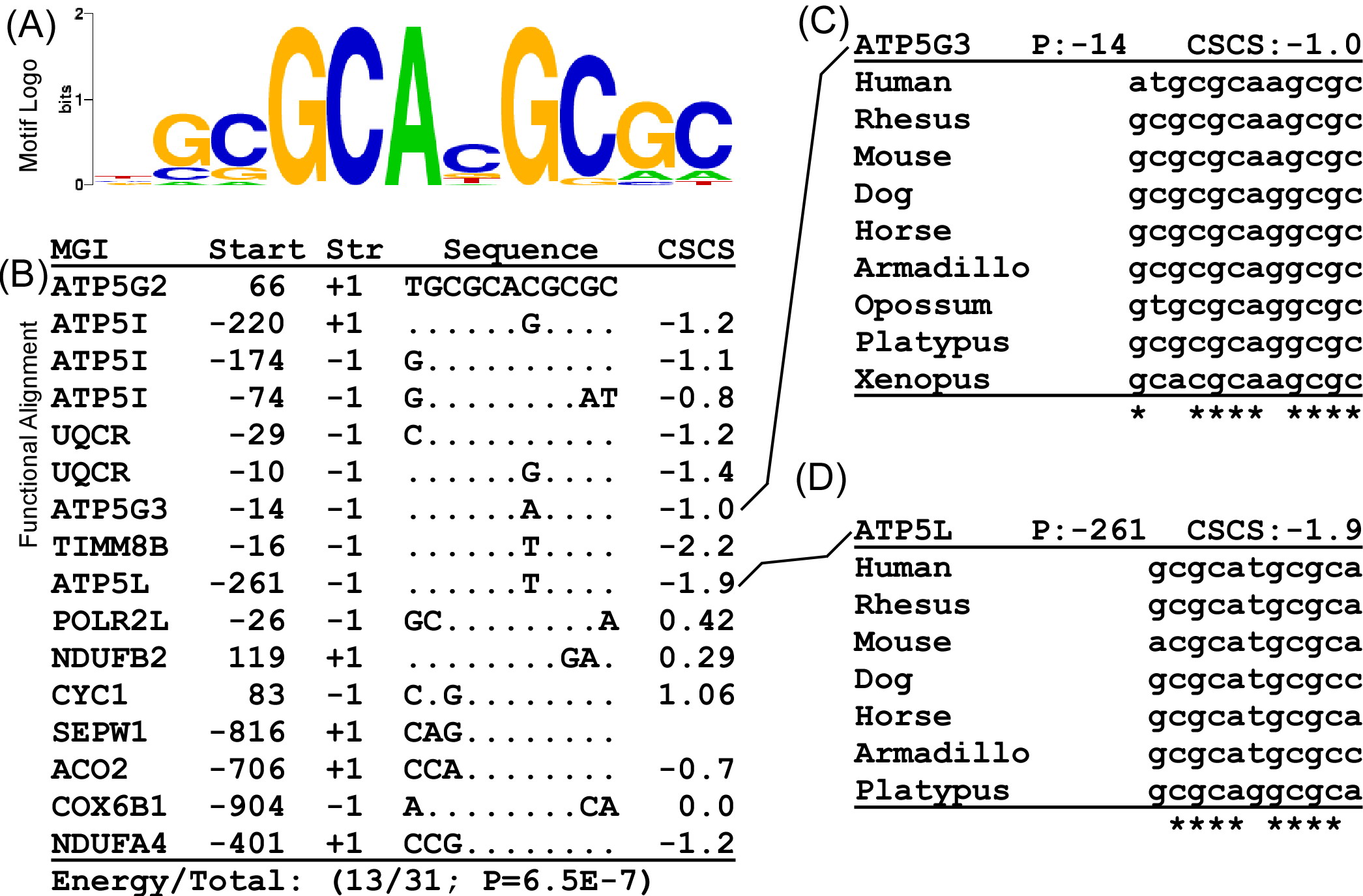

Supplement: Additional file 4 — A cis-element enriched in promoters driving expression of genes in the energy generation module. A sample cis-element enriched in promoters driving the expression of genes in the energy generation module. (A) The motif logo. (B) The cross-promoter alignment. Columns, from left to right, give the MGI symbol, start position, strand relative to the transcription start site (+1), consensus sequence (shown on top), and cross-species conservation score (CSCS; negative indicates strong phylogenetic conservation). (C-D) Sample phylogenetic alignments for occurrences in ATP5G3 (CSCS = -1.0, C) and ATP5L (CSCS = -1.9, D). [file 1755-8794-2-31-S4.jpeg]

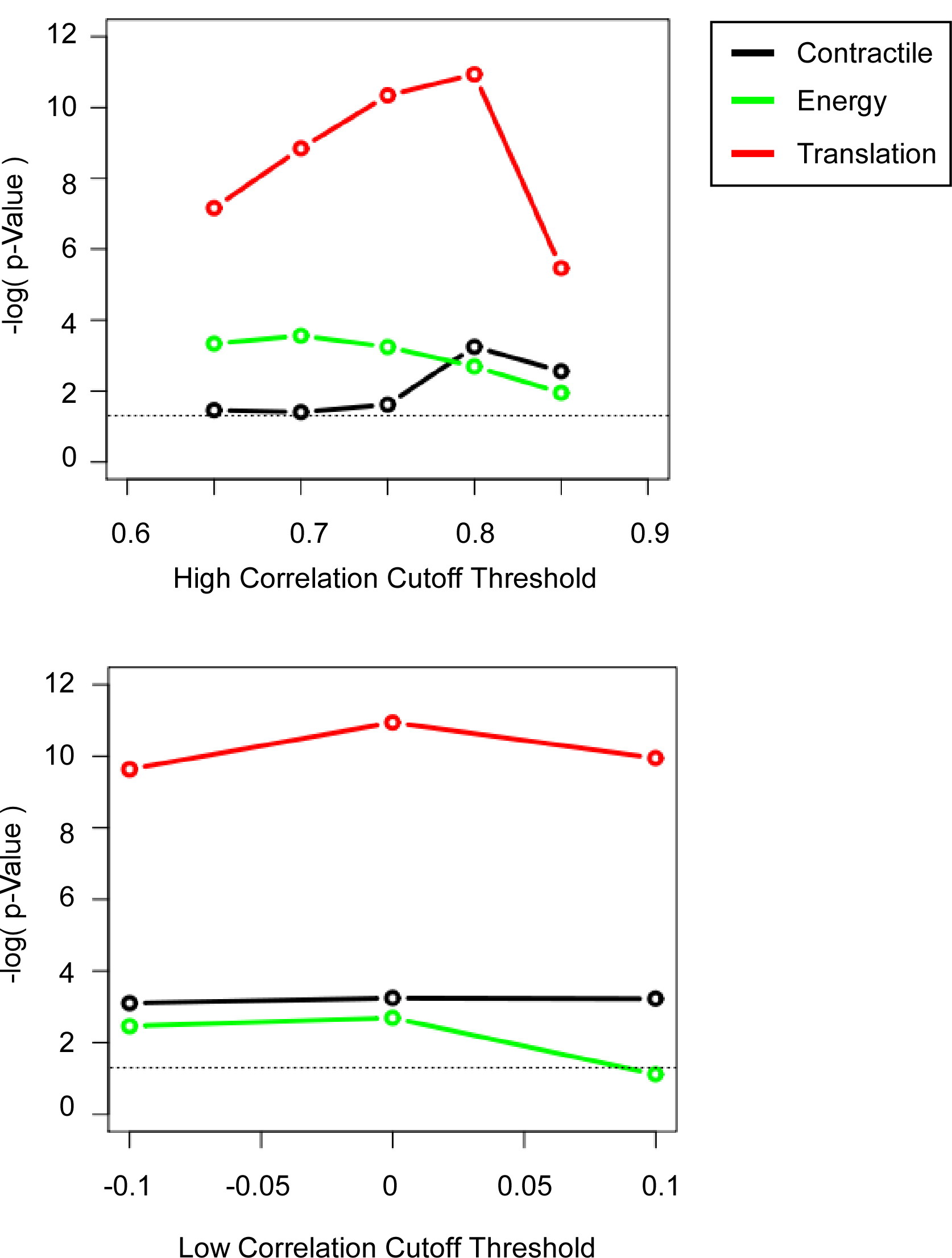

Supplement: Additional file 5 — Plot of negative log of p-value as a function of high and low correlation cutoff thresholds. Negative log of p-values represent the enrichment of the pattern of motifs in promoter sequences that drive highly correlated ("high") expression with respect to uncorrelated ("low") expression. Values along the horizontal axis are the Pearson correlation above which genes are classified as "highly" correlated (between 0.65 and 0.85; top), or below which genes are classified as having a "low" correlation (bottom; -0.10 to 0.10), as described in Methods. P-values are plotted separately for classification based on the contractile (black), energy generation (green) and protein translation (red) module. High correlation thresholds (top) are plotted with respect to a constant low correlation threshold (R = 0.0). Low correlation thresholds (bottom) are plotted using a constant high correlation threshold (R = 0.80). The dotted line represents a p-value cutoff of 0.05, indicating that 23 of 24 parameter combinations are statistically significant at this cutoff threshold (p < 0.05). This plot demonstrates that the results of the naïve Bayes classification are robust to changes in these parameters. [file 1755-8794-2-31-S5.jpeg]
